# Assessment of progesterone levels on the day of pregnancy test determination: A novel concept toward individualized luteal phase support

**DOI:** 10.3389/fendo.2023.1090105

**Published:** 2023-02-01

**Authors:** A. Racca, M. Alvarez, S. Garcia Martinez, I. Rodriguez, I. Gonzalez-Foruria, NP. Polyzos, B. Coroleu

**Affiliations:** ^1^ Department of Obstetrics Gynecology and Reproductive Medicine, Dexeus University Hospital, Barcelona, Spain; ^2^ Facultad de Medicina Universitat de Vic-Universitat Central de Catalunya, Vic, Spain; ^3^ Faculty of Medicine and Health Sciences, Ghent University, Ghent, Belgium

**Keywords:** luteal phase support, artificially prepared endometrium, hCG and progesterone assessment, individualization of luteal phase, progesterone supplementation, live birth rate

## Abstract

**Research question:**

The main objective of the study is to define the optimal trade-off progesterone (P4) values on the day of embryo transfer (ET), to identify low P4-human chorionic gonadotropin (hCG), and to establish whether P4 supplementation started on the hCG day can increase the success rate of the frozen embryo transfer (FET) cycle.

**Design:**

A single-center, cohort, retrospective study with 664 hormone replacement therapy (HRT)-FET cycles analyzed female patients who received vaginal 600 mg/day of P4 starting from 6 days before the FET, had normal P4 values on the day before ET, and whose P4 on the day of the pregnancy test was assessed.

**Results:**

Of the 664 cycles, 69.6% of cycles showed P4 **≥** 10.6 ng/ml, while 30.4% showed P4 < 10.6 ng/ml on the day of the hCG. Of the 411 chemical pregnancies detected, 71.8% had P4-hCG **≥** 10.6 ng/ml (group A), while 28.2% had P4-hCG < 10.6 ng/ml. Of the cycles with P4-hCG < 10.6 ng/ml, 64.7% (group B) were supplemented with a higher dose of vaginal P4 (1,000 mg/day), while 35.3% (group C) were maintained on the same dose of vaginal micronized P4. The live birth rate was 71.9%, 96%, and 7.3% for groups A, B, and C, respectively.

**Conclusion:**

The likelihood to detect P4-hCG < 10.6 ng/ml decreased as the level of serum P4 the day before ET increased. The live birth rate (LBR) was shown to be significantly lower when P4 was low and not supplemented.

## Introduction

Approximately 2.4 million assisted reproductive technology (ART) procedures per year are reported worldwide with 500, 000 babies born per year (1). Owing to the improvement in embryo culture, the introduction of vitrification, and the evidence supporting the advantage of freezing in some (elective or non-elective) situations (2), the ratio of frozen embryo transfer (FET) to fresh embryo transfer procedures has increased dramatically over the past few years from 28% to 44.1% (2010–2016) and from 22.9% to 69.4% (2010 –2017), respectively, in Europe and the USA (3) “ART Success Rates | CDC”, 2020).

Although FET can be planned with either an artificial cycle (hormone replacement therapy (HRT) FET) or a natural cycle (NC FET) (4), no approach has demonstrated inferiority regarding clinical outcomes (5, 6). HRT FET cycles are mostly used due to their practicality (minimal cycle monitoring and easy scheduling). The two main protagonists of the HRT cycle are estrogens (E_2_) and p rogesterone (P4). For the E_2_ priming of the endometrium, it has not been demonstrated that serum oestradiol levels are associated with the cycle outcomes (7, 8); this is not the case for serum P4, where multiple studies have confirmed its crucial role in the establishment and maintenance of the pregnancy (9, 10).

Progesterone can be administered in different ways: vaginally, intramuscularly, rectally, orally, or subcutaneously. The most used is the vaginal route; however, the subcutaneous way is becoming more and more popular (11, 12). Intramuscular P4 (IM P4) is still widely used in some parts of the world; however, in Europe, it is quite unpopular. Progesterone has two key features: one is that pharmacokinetics depends on the route of administration and consequently the serum values (13); the other one is the daily variability (14, 15). Despite the high variability of P4, all the recent studies published on luteal serum P4 showed that low serum levels of P4 (below 9–10 ng/ml) are associated with worse cycle outcomes (10, 16–20), independently from the day of the assessment.

Encouraging data collected in a previous prospective study from our group showed the possibility to rescue the FET cycle when the P4 value was found to be <10.6 ng/ml on the day before the FET by supplementing progesterone on the same day (18).

In light of the above, P4 assessment in the luteal phase is of greatest importance as well as the possibility to supplement the deficient cycle with more P4 [progesterone supplementation (PS)], the so-called “rescue protocol” (10, 16, 18, 21, 22).

Nonetheless, until now, all the studies have concentrated on a single measurement of P4, the day before or the day of FET itself. However, considering the important daily variability of the P4 values, and the possibility to individualize the luteal phase (iLP), a single measurement may not be sufficient to establish the appropriateness of the entire luteal phase. Therefore, the aims of the present study were as follows: first, to define optimal trade-off P4 values on the day of embryo transfer (ET) and to identify low P4 values on the day of pregnancy test; second, to investigate the clinical pregnancy rate (CPR) and live birth rate (LBR) in patients with three different conditions, namely, normal P4 not supplemented, low P4 supplemented, and low P4 not supplemented; and third, to determine whether P4 supplementation on the day of pregnancy test improves clinical pregnancy and live birth rate.

## Methods

### Study setting

This is an exploratory, cohort, retrospective, single-center study performed at a university-affiliated fertility center between January 2018 and June 2020. Patients undergoing FET with artificial preparation of the endometrium (ET-HRT) who had adequate values of P4 (**≥**10.6 ng/ml) the day before ET were included. The entire study group underwent a second assessment of P4 on the day of the pregnancy test, and in case of positive human chorionic gonadotropin (hCG), whenever P4 was found to be below 10.6 ng/ml, additional vaginal P4 was supplemented on the same day. The study was approved by the institutional review board of Dexeus Mujer (approval number: 072020102604).

### Selection of study population

Frozen heterologous ET-HRT (het-ET), homologous ET-HRT (hom-FET), and euploid ET-HRT (eu-FET) after preimplantation genetic testing for aneuploidies (PGT-A) *in vitro* fertilization (IVF) cycles were considered for the analysis.

Patients with known uterine abnormalities or mosaic embryos and with serum P4 extraction taken after 11 a.m. were excluded.

### Study protocol

Embryos were kept in culture in a time-lapse incubator, single-step culture media (LifeGlobal^®^, Paramus, NJ, USA), with 5% oxygen concentration, as described previously (17). Embryos were transferred at the stage of the blastocyst. When performed, the PGT-A procedure was carried out as previously described (23). Embryos were biopsied and vitrified on days 5 and 6 of development. Genetic testing on the embryo was performed by array comparative genomic hybridization (a-CGH) using accessible kits and software (SurePlex^®^ DNA Amplification System, 24Sure^®^ Microarray Pack, BlueFuseMulti^®^, Illumina^®^) following the manufacturer’s instructions.

### Endometrial preparation

Patients received 2 mg/8 h of estrogen valerate (E2) (Progynova^®^, Schering, Berlin, Germany) for 10–12 days and subsequently underwent a vaginal ultrasound to determine the thickness and the morphology of the endometrium. In case of adequateness of the endometrium (>7 mm and trilaminar), vaginal micronized P (Utrogestan®) treatment (200 mg/8 h) was started at night (D0). Embryo transfer was planned on the sixth day of P4 supplementation (D6). The first assessment of the serum P4 level was performed for all patients included in the analysis on the day prior to the FET (D4) between 8 and 11 a.m., using an electrochemiluminescence immunoassay (Cobas^®^ e-41 analyzer; Roche Diagnostics, Mannheim, Germany). For P4, the lower limit of detection was 0.05 ng/ml (intra- and inter- assay variations of 1.2%–11.8% and 3.6%–23.6%, respectively).

**Figure 1:**
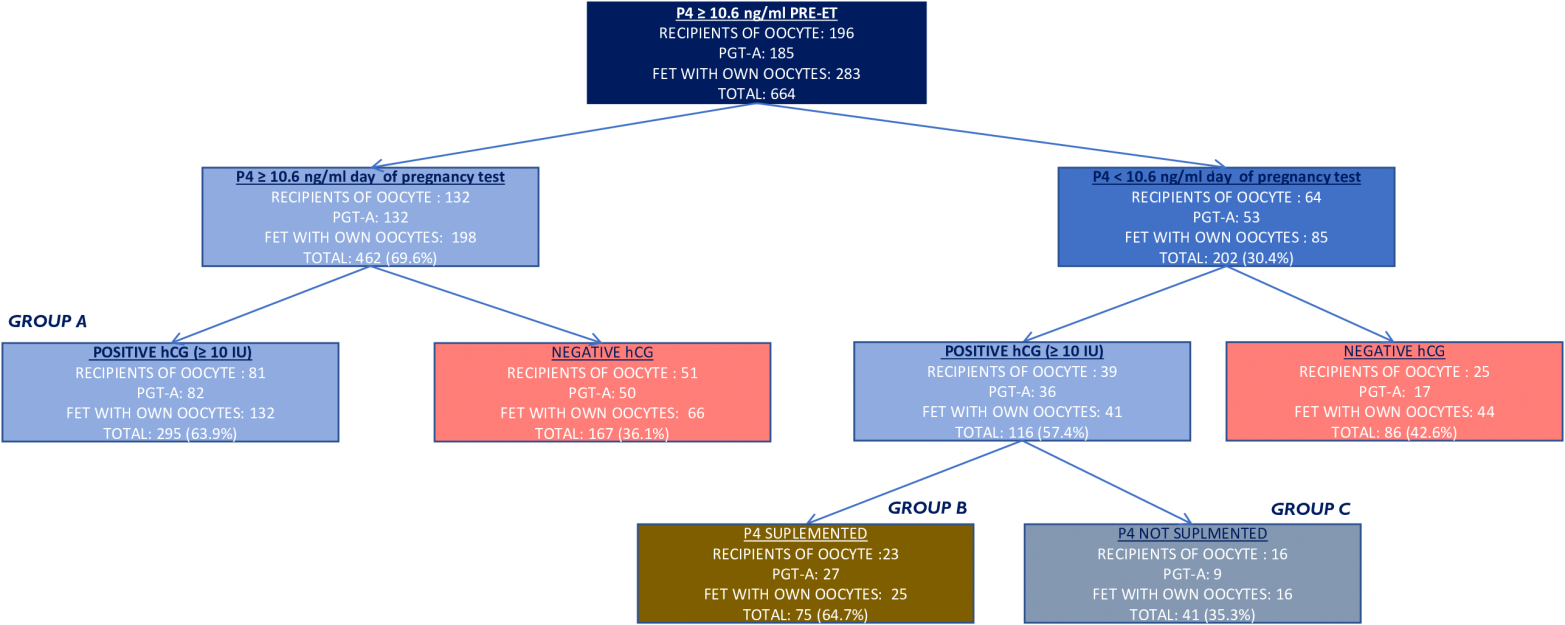
Flow chart of the study population and results. In red are the cycles excluded from the primary outcome. In gold is the supplementation on the day of hCG assessment. Positive hCG was considered when ≥ 10 IU. hCG, human chorionic gonadotropin.

Only cycles with P4 levels **≥**10.6 ng/ml on the day prior to FET were included in the study. Then, P4 was analyzed on the β -hCG day, 10 days after the FET (P4-β- hCG). Patients with P4-β- hCG ≥ 10.6 ng/ml continued the luteal phase support with vaginal P4 200 mg/8 h (group A); patients with P4-β- hCG < 10.6 ng/ml were either supplemented with P4 by means of increased dose (1,000 mg/day, divided into 400 mg in the morning, 200 mg in the afternoon, and 400 mg in the evening) of daily vaginal P4 (group B) or maintained on the same dose of vaginal P4 (not supplemented) (group C), as described in **Figure 1**. The P4-β- hCG cutoff value was stated at 10.6 ng/ml according to Gaggiotti-Marre et al. (22) where the miscarriage rate was higher for patients with low P4 on the day before ET and the live birth rate was lower in the same group.

Treatment discontinuation was usually individualized at around the 10th week of pregnancy, but not rigorously defined.

### Outcomes

The primary outcome was to define the optimal trade-off P4 values on the day of ET to identify low p-values on the day of the pregnancy test.

The secondary outcomes of the present study were to investigate CPR and LBR in patients with three different conditions: normal P4 not supplemented, low P4 supplemented, and low P4 not supplemented. Furthermore, this study aimed to determine whether P4 supplementation on the day of pregnancy test improves clinical pregnancy and live birth rate.

CPR was defined as the presence of a vital embryo at 7 weeks of amenorrhea, LBR was described as the delivery of at least one live- born neonate per transfer, and miscarriage rate (MR) was stated as a clinical pregnancy loss before week 23 of gestation (24).

### Statistical analysis

Mean and standard deviation were used as continuous variables, while frequencies and percentages were used as categorical variables. Continuous variables were compared through Student’s t- test or ANOVA, while the chi-squared test was used to compare categorical variables.

A receiver operating characteristic (ROC) curve and Youden’s index were used to find a pre-transfer P4 cutoff point that maximizes sensitivity and specificity for diagnosing low progesterone on the day of pregnancy test assessment. To investigate CPR and LBR in three different conditions, an exploratory comparison between groups A, B, and C was performed. To investigate the effect of P4 supplementation on clinical pregnancy and live birth rate, a between- group comparison (patients with low P4 on the day of pregnancy test receiving versus not receiving P4 supplementation) was performed. For all group comparisons, the analysis was performed in two steps: first, with the entire cohort of pregnant patients (hCG **≥** 10 IU) and second, only with the good prognosis pregnancy (hCG **≥** 100 IU) to control for the hCG levels that were significantly higher in group B compared to groups A and C. All the analyses were exploratory. No formal sample size calculation was performed.

All tests were two- tailed, and a p-value of <0.05 was considered statistically significant. Statistical analyses were performed with IBM^©^ SPSS^©^ Statistics v22.

## Results

### Patients characteristics

A total of 664 frozen embryo cycles, corresponding to 554 patients, performed under HRT where 200 mg micronized P4 was administered twice a day, and with P4 value **≥**10.6 ng/ml on the day before ET, were analyzed, as described in **Figure 1** (196 het-ET, 283 hom-FET, and 185 eu-FET).

In **Table 1**, all the 664 eligible cycles are divided into two groups given P4 levels on the day of pregnancy test assessment, above or under the threshold of 10.6 ng/ml. Specifically, 462 (69.6%) cycles showed appropriate (**≥**10.6 ng/ml) P4 values, while 202 (30.4%) showed inadequate (<10.6 ng/ml) levels of P4 on the day of the hCG measurement. None of the variables investigated differed in the two groups aside from the serum levels of P4 before the ET, being lower in the group with inadequate levels of P4 on the day of pregnancy test measurement (13.3 *vs.* 15.6, p < 0.001).

**Table 1:** Baseline characteristics between normal P4 and low P4 on the day of hCG.

	Progesterone ≥ 10.6 ng/ml N: 462 (69.6%)	Progesterone < 10.6 ng/ml N: 202 (30.4%)	p
Age oocyte (years)	33.3 ± 6.2	33.1 ± 6.2	0.685
BMI	24.4 ± 4	25.1 ± 4.8	0.122
**P4 day before transfer (ng/ml)**	**15.6 ± 5.3**	**13.3 ± 3.9**	**<0.001**
Endometrial thickness (mm)	10.5 ± 1.9	10.6 ± 1.9	0.911
Number of embryos transferred	1.08 ± 0.3	1.04 ± 0.2	0.066
Good-quality embryo transferred	0.6 ± 0.5	0.6 ± 0.5	0.943
P4 day β-hCG (ng/ml)	16.3 ± 6.6	8.9 ± 1.5	
hCG	156.8 ± 227.3	159.8 ± 260	0.881
hCG ≥ 10	63.9% (295)	57.4% (116)	0.117

In bold are statistically significant values. All values are mean ± SD.

BMI, body mass index; P4, progesterone.

#### Progesterone levels on the day of pregnancy test in the pregnant group

Out of 664 cycles, there were 411 (61.9%) positive hCG (hCG **≥** 10 IU). The second part of the analysis focused on this group. **Table 2** shows a division into two subgroups according to the positive (411/664, 61.9%) or negative hCG (253/664, 38.1%). The main difference between the two groups was body mass index (BMI) (24.9 *vs.* 23.9, p = 0.014), mean number of good quality embryos transferred (0.7 *vs.* 0.5, p < 0.001), and P4 levels on the day of the pregnancy test, being all significantly higher in favor of the pregnant group.

**Table 2:** Baseline characteristics between hCG positive (hCG ≥ 10 IU) and. hCG negative (hCG < 10 IU).

	hCG ≥ 10 IU N: 411 (61.9%)	hCG< 10 IU N: 253 (38.1%)	p
Age oocyte (years)	32.9 ± 6.1	33.7 ± 6.4	0.141
**BMI**	**24.9 ± 4.4**	**23.9 ± 3.9**	**0.011**
PGT-A	118/411 (28.7%)	67/253 (26.5%)	0.824
P4 day before transfer (ng/ml)	15 ± 5.2	14.7 ± 4.9	0.395
Endometrial thickness (mm)	10.6 ± 1.9	10.3 ± 1.9	0.094
Number of embryos transferred	1.1 ± 0.3	1.1 ± 0.3	0.644
**Good-quality embryo transferred**	**0.7 ± 0.5**	**0.5 ± 0.5**	**<0.001**
Score transfer	9.3 ± 1.1	9.2 ± 1.2	0.220
**P4 day hCG (ng/ml)**	**14.7 ± 7.3**	**13 ± 4.7**	**<0.001**

In bold are statistically significant values. All values are mean ± SD.

BMI, body mass index; PGT-A, preimplantation genetic testing for aneuploidies; P4, progesterone.

#### Definition of an optimal pre-ET P4 cutoff point to identify low P4 values on the day of the pregnancy test

Results showed that the likelihood to detect P4-hCG < 10.6 ng/ml decreases as the level of serum P4 the day before ET increases.

**Figure 2:**
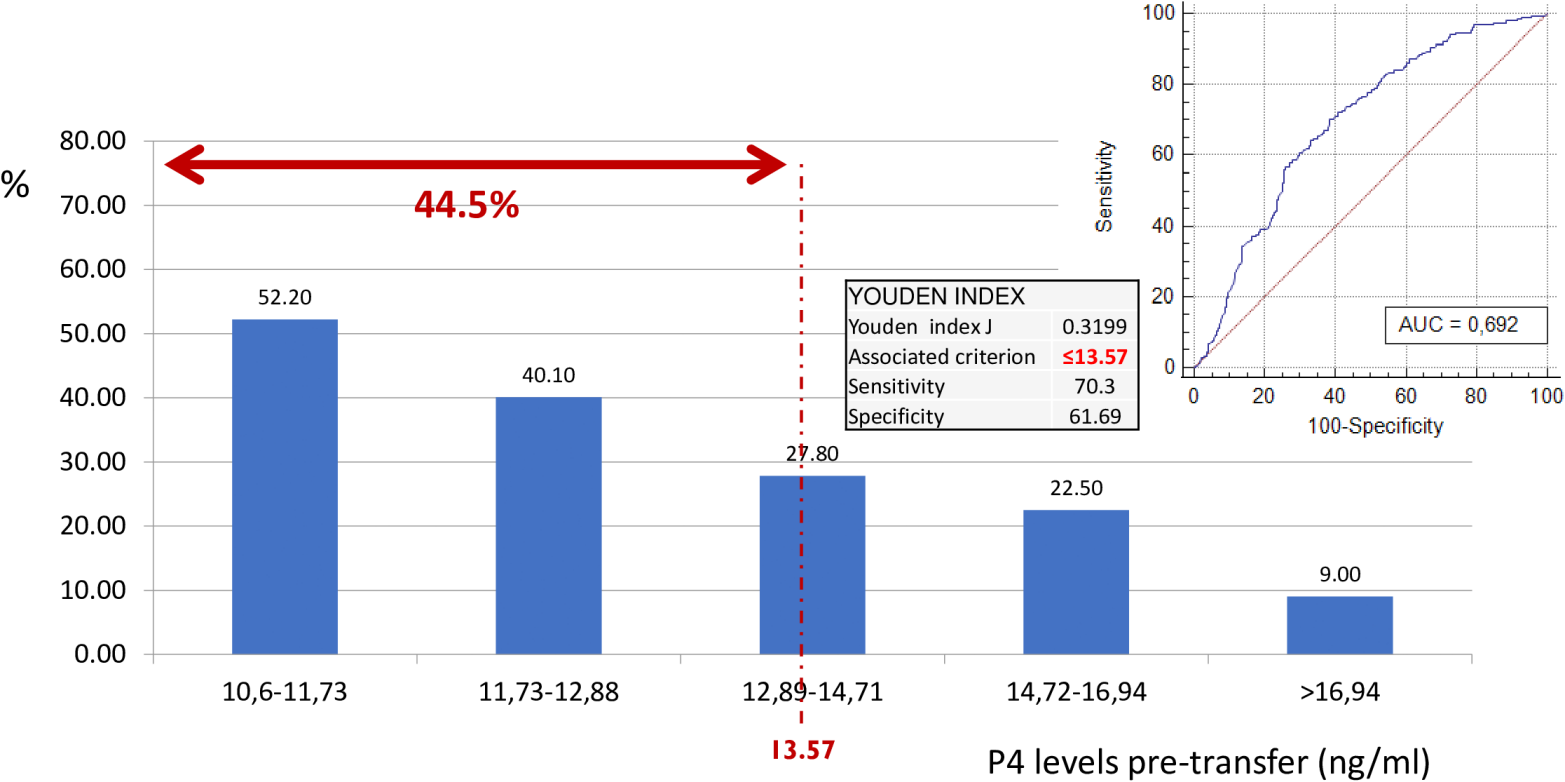
Probability of having progesterone levels < 10.6 on day of pregnancy test according to pre-transfer progesterone levels. ROC curve. Classification of low P on the day of hCG. Considering the P4 value on the day before ET. As a result of this ROC curve, a cutoff point of 13.57 ng/ml was selected with specific sensitivity and specificity values. Therefore, 44.5% of the cycles with progesterone ≤ 1 3.57 ng/ml, despite being “high”, have a low progesterone value on the day of hCG. ROC, receiver operating characteristic; hCG, human chorionic gonadotropin; P4, progesterone; ET, embryo transfer.

As the primary outcome, an optimal trade-off of P4 values on the day of ET to identify low P4 values on the day of the pregnancy test was defined. A ROC analysis (**Figure 2**) showed an area under the curve (AUC) of 0.69 (sensitivity 70.3 and specificity 61.7), indicating that if P4 is measured once (on the day before ET), a value of 13.6 ng/ml is required to assume that P4 will most likely stay above the threshold of 10.6 ng/ml within the first 10 days of the luteal phase.

#### Exploratory comparison between patients with normal P4 values and low P4 values, receiving or not receiving P4 supplementation on the day of pregnancy test

In **Table 3**, the population of pregnant women was divided according to P4-hCG and P4 supplementation or not in case the P4 was lower than 10.6 ng/ml. The three groups differed on BMI (24.6 *vs.* 25.9 *vs.* 26.7, p = 0.021), mean P4 on the day before ET (15.7 *vs.* 13.9 *vs.* 12.6, p < 0.001), mean P4 on the day of the pregnancy test (16.9 *vs.* 9.3 *vs.* 8.5, p > 0.001), and mean hCG value (245.3 *vs.* 327.8 *vs.* 185.8, p = 0.009), where group B showed significantly higher hCG values than groups A and C.

**Table 3:** Only hCG- positive cycles (hCG ≥ 10 IU); group division is based on the levels of P4 on the day of pregnancy test and supplementation or no supplementation.

	A (N: 295)	B (N: 75)	C (N: 41)	p
Age oocyte (years)	33.1 ± 6.1	32.7 ± 5.5	32.5 ± 6.6	0.797
**BMI**	**24.6 ± 4.1**	**25.9 ± 5.1**	**26.7 ± 5.3**	**0.021**
PGT-A	82/256 (27.8%)	27/75 (36%)	9/19 (22%)	0.215
**P4 day before transfer (ng/ml)**	**15.7 ± 5.1**	**13.9 ± 5.9**	**12.6 ± 1.6**	**<0.001**
Endometrial thickness (mm)	10.6 ± 1.9	10.6 ± 1.8	10.4 ± 2.1	0.740
Number of embryos transferred	1.08 ± 0.3	1.03 ± 0.2	1.05 ± 0.2	0.251
Good-quality embryo transferred	0.7 ± 0.5	0.7 ± 0.5	0.6 ± 0.5	0.315
Score transfer	9.3 ± 1.2	9.6 ± 0.9	8.9 ± 1.3	0.014
**P4 day hCG (ng/ml)**	**16.9 ± 7.5**	**9.3 ± 1.3**	**8.5 ± 1.7**	**<0.001**
**hCG**	**245.3 ± 243.6**	**327.8 ± 278.7**	**185.8 ± 296.9**	**0.009**

Group A, P4 ≥ 10.6 ng/ml (no supplementation); group B, P4 < 10.6 ng/ml (supplemented); group C, P4 < 10.6 ng/ml (no supplementation). All values are mean ± SD, and p- values refer to a three-group comparison.

hCG, human chorionic gonadotropin; BMI, body mass index; PGT-A, preimplantation genetic testing for aneuploidies; P4, progesterone. In bold are statistically significant values.

#### P4 levels on the day of pregnancy test with possible P4 supplementation and clinical outcomes (for hCG positive)

Of the 411 chemical pregnancies assessed (hCG **≥** 10 IU), 295 (71.8%) had P4-hCG **≥** 10.6 ng/ml again, while 116 (28.2%) had P4-hCG < 10.6 ng/ml on the same day. Of 116 (64.7%) cycles with an inadequate level of P4 on the day of the pregnancy test, 75 (64.7%) were supplemented with a higher dose of vaginal P4 (1,000 mg/day), while 41 out of 116 (35.3%) were maintained on the same dose of vaginal micronized P4. Eventually, clinical pregnancy and LBR were compared between the three groups: groups A, B, and C.

**Figure 3:**
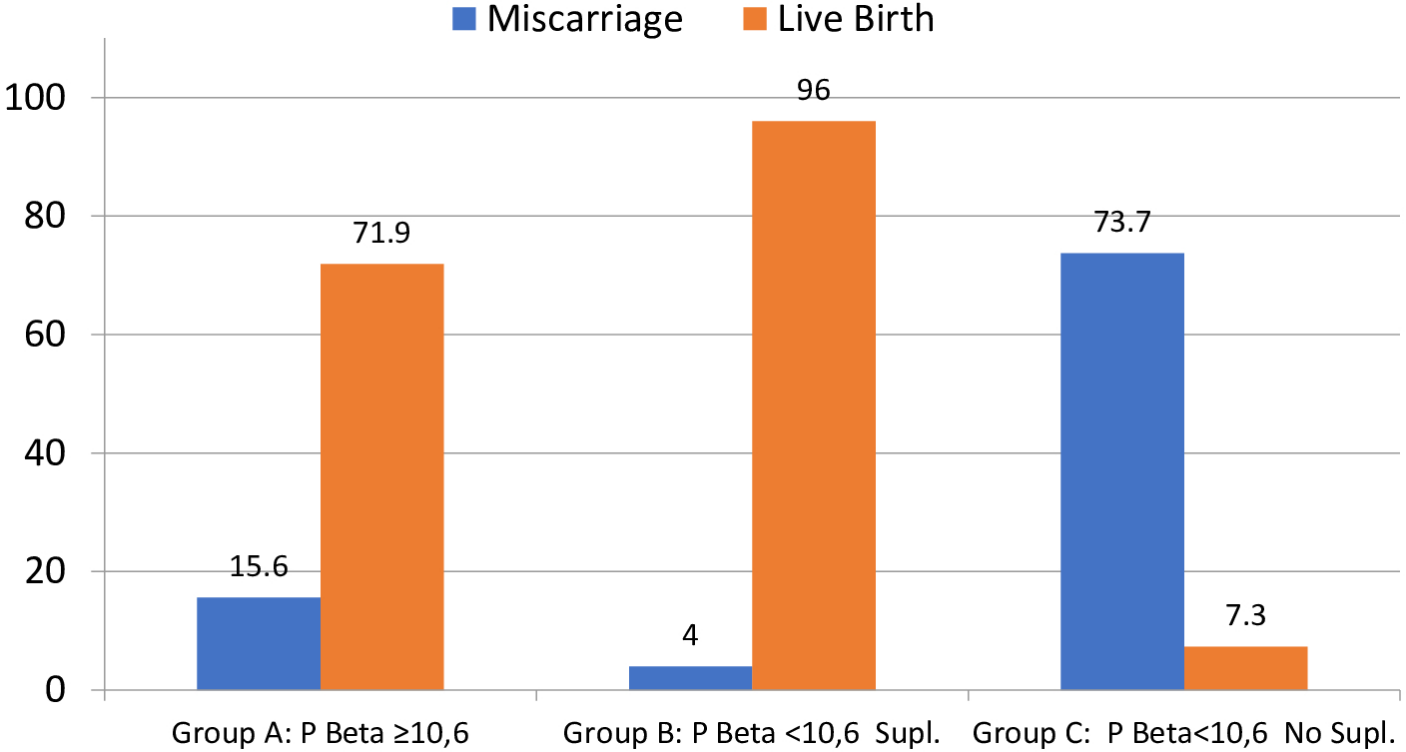
Percentage of miscarriage and live birth per positive hCG (≥10 IU). Note that groups A and C do not reach 100% because group A has three VIG (>12 weeks) and 1 still birth (malformation) and group C has two VIG (>12 weeks). VIG, voluntary interruption of gestation; hCG, human chorionic gonadotropin. Miscarriage p < 0.0001; live birth p < 0.0001.

**Figure 4:**
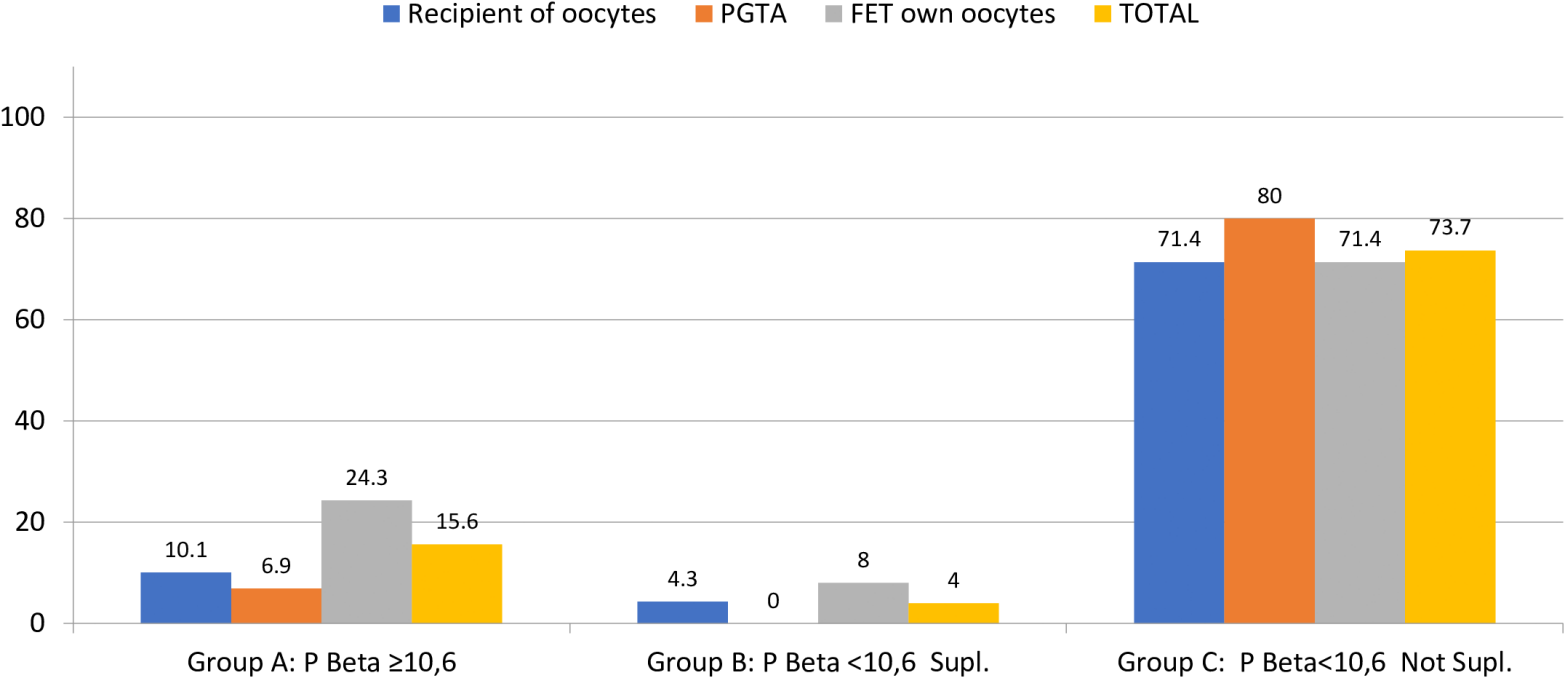
Miscarriage/pregnancy in the different groups of patients (per progesterone).

Clinical pregnancy was significantly lower for group C, compared to groups B and A (46 *vs.* 100 *vs.* 87%), and the same was shown for live birth also significantly lower in group C (mean LBR 7.3%) compared to group A (mean LBR 71.9%) and group B (mean LBR 96%), regardless of the FET-IVF technique used (recipients of oocytes, PGT-A, or FET with own oocytes, **Table 4**; **Figures 3**, **4**).

**Table 4:** Clinical outcomes per group of progesterone and supplementation or not analyzed for all the pregnancies considered with hCG ≥ 10 IU.

	A (N = 295)	B (N = 75)	C (N = 41)	p-Value
Mean hCG	245.3 ± 243.6	327.8 ± 278.7	185.8 ± 296.9	0.009
Clinical pregnancy				
Recipients of oocytes (%)	69/81 (85.2)	23/23 (100)	7/16 (43.8)	<0.001
PGT-A (%)	72/82 (87.8)	27/27 (100)	5/9 (55.6)	0.002
FET with own oocytes (%)	115/132 (87.1)	25/25 (100)	7/16 (43.8)	<0.001
Total (%)	256/295 (86.8)	75/75 (100)	19/41 (46.3)	<0.001
Live birth				
Recipients of oocytes (%)	61/81 (75.3)	22/23 (95.7)	1/16 (6.3)	<0.001
PGT-A (%)	65/82 (79.3)	27/27 (100)	1/9 (11.1)	<0.001
FET with own oocytes (%)	86/132 (65.2)	23/25 (92)	1/16 (6.3)	<0.001
Total (%)	212/295 (71.9)	72/75 (96)	3/41 (7.3)	<0.001
Miscarriage				
Recipients of oocytes (%)	7/69 (10.1)	1/23 (4.3)	5/7 (71.4)	<0.001
PGT-A (%)	5/72 (6.9)	0/27 (0)	4/5 (80)	<0.001
FET with own oocytes (%)	28/115 (24.3)	2/25 (8)	5/7 (71.4)	<0.001
Total (%)	40/256 (15.6)	3/75 (4)	14/19 (73.7)	<0.001

Group A, P4 ≥ 10.6 ng/ml (no supplementation); group B, P4 < 10.6 ng/ml (supplemented); group C, P4 < 10.6 ng/ml (no supplementation). Values are reported as crude numbers and (%). p- values refer to a three-group comparison.

hCG, human chorionic gonadotropin; PGT-A, preimplantation genetic testing for aneuploidies; FET, frozen embryo transfer.

#### P levels on the day of pregnancy test with possible P4 supplementation and clinical outcomes (for hCG ≥ 100 IU)

Due to the significantly lower values of hCG in group C and the arbitral decision to supplement P4 when necessary, and in the intent to better understand the real effect of P4 supplementation, in the third part of the analysis, only the good prognosis pregnancies were considered (according to hCG levels **≥** 100 IU) (**Table 5**). By excluding all the pregnancies with hCG < 100 IU (first hCG assessment, 10 days after ET), the three groups became smaller: A with N = 214, B with N = 64, and C with N = 19. T he mean (SD) hCG values were respectively 316.5 (251.1) IU, 372.8 (277.8) IU, and 349.1 (377.6) IU (p = 0.318) for groups A, B, and C.

**Table 5:** Clinical outcomes per group of progesterone and supplementation or not for all the pregnancies with first hCG value ≥ 100 IU.

	A (N = 214)	B (N = 64)	C (N = 19)	p-value
Mean hCG	316.5 ± 251.1	372.8 ± 277.8	349.1 ± 377.6	0.318
Clinical pregnancy				
Recipients of oocytes (%)	57/60 (95)	19/19 (100)	6/8 (75)	0.035
PGT-A (%)	59/61 (96.7)	26/26 (100)	2/2 (100)	0.625
FET with own oocytes (%)	89/93 (95.7)	19/19 (100)	6/9 (66.7)	0.001
Total (%)	205/214 (95.8)	64/64 (100)	14/19 (73.7)	<0.001
Live birth				
Recipients of oocytes (%)	51/60 (85)	19/19 (100)	1/8 (12.5)	<0.001
PGT-A (%)	54/61 (88.5)	26/26 (100)	1/2 (50)	0.028
FET with own oocytes (%)	71/93 (76.3)	17/19 (89.5)	1/9 (11.1)	<0.001
Total (%)	176/214 (82.2)	62/64 (96.9)	3/19 (15.8)	<0.001
Miscarriage				
Recipients of oocytes (%)	6/57 (10.5)	0/19 (0)	5/6 (83.3)	<0.001
PGT-A (%)	4/59 (6.8)	0/26 (0)	1/2 (50)	0.051
FET with own oocytes (%)	17/89 (19.1)	2/19 (10.5)	4/6 (66.7)	0.001
Total (%)	27/205 (13.2)	2/64 (3.1)	10/14 (71.4)	<0.001

Group A, P4 ≥ 10.6 ng/ml (no supplementation); group B, P4 < 10.6 ng/ml (supplemented); group C, P4 < 10.6 ng/ml (no supplementation). Values are reported as crude numbers and (%). p- Values refer to three-group comparisons.

hCG, human chorionic gonadotropin; PGT-A, preimplantation genetic testing for aneuploidies; FET, frozen embryo transfer.

Clinical pregnancy was respectively 74%, 100%, and 96% for groups C, B, and A, while the live birth rates were 15.8%, 96.9%, and 82.2%, respectively, for groups C, B, and A.

#### The effect of P4 supplementation for patients with low P4 values on the day of pregnancy test

Within the cohort of cycles with low P4 values on the day of the pregnancy test, 75 cycles were supplemented and 41 were not supplemented with extra P4. Better outcomes were found in the group that received P4 supplementation, in terms of CPR and LBR of 100%*vs.* 46.7%, respectively, for supplemented *vs.* not supplemented, and LBR was 96% *vs.* 7.3%, respectively, for supplemented *vs.* not supplemented (p > 0.001).

## Discussion

To our knowledge, this is the first study demonstrating that P4 levels should be assessed more than once during the luteal phase. Based on our findings, performing a second assessment on those cycles with initially normal P4 values shows that approximately one-third of them resulted in abnormally low P4 values. Furthermore, we showed that the higher the P4 levels on the day before ET, the lower the likelihood of inadequate serum levels of P4 on the day of pregnancy test assessment. The clinical relevance of such a finding is that a second P4 assessment permits a better follow-up of the serum P4 and allows the “rescue protocol” at different time points of the luteal phase, thus increasing the live birth rate.

In fact, when looking at the ability of the first P4 assessment to predict the second P4 assessment, an area under the ROC curve of 0.69 resulted, indicating that the lowest level can be statistically defined as acceptable discrimination (25). However, from a clinical perspective, a second assessment may be always preferable, given the relatively low costs and invasiveness of the procedure.

Focusing on the secondary outcome, the results of the present study demonstrate a strong association between serum P4 on the day of pregnancy tests and live birth rates. In fact, in those FET-HRT cycles where P4 could be kept above 10.6 ng/ml throughout the entire luteal phase, the live birth rate was higher. A significantly lower LBR was found when no progesterone supplementation was performed in case P4-hCG < 10.6 ng/ml, independently on the first hCG value. Because hCG was significantly higher in group B compared to groups A and C, the specific analysis of the cycle outcomes was performed considering two groups (the entire population of positive hCG (≥10 IU) and only the hCG **≥** 100 IU). One could postulate that the arbitral decision of adding P4 was taken only for good prognosis patients, thus biasing the results of the study. However, when considering only the good prognostic pregnancies, the results remained the same, confirming that the biggest influence on the outcome (LBR) is driven by adequate values of P4 for the whole luteal phase.

To summarize, the clinical “take-home message” is that we cannot rely on a single assessment of P4 in the early luteal phase. Therefore, assessing P4 also on the day of the pregnancy test is of utmost importance to establish the adequacy of the P4 value in the late luteal phase and possibly enhance the supplementation, with the result of increasing live birth rate. In fact, the luteal phase can be seen as a “window of opportunity” where repeating the assessment of P4 on the day of the hCG gives us the possibility to still revert to a deficient cycle and be on time.

The current results are in line with the outcomes of the research of the last years, where the main message is the importance of luteal P4 evaluation in artificially prepared endometrium cycles (10, 16, 18, 21, 22). Although having different timing in the P4 assessment, quite impressively, all the previous studies nearly showed the same outcome, having higher values of luteal P4 (>9 ng/ml) related to higher outcomes rate. More specifically, the consistency of these results goes beyond the timing in which progesterone was evaluated (day of FET, day before FET, or 11 days after FET, respectively, for 10, 16, 18, 19, 22). In fact, the common feature of all the results was that whenever P4 was in the lower limit, the cycle outcomes were hindered. Although nowadays many clinics are performing FET in the natural or modified natural cycle due to the evidence of the latest years supporting the role of the corpus luteum (von 26), a great fraction of FET cycles are performed in an artificial cycle, especially due to the advantages of least cycle monitoring, easy scheduling, and wider applicability even to women without ovarian cycle. The artificial cycle is widely used and mostly with the scheme of 10–2 days of oral estrogens followed by vaginal P4 administered with a common dose of 200 mg three to four times a day (4). As determined by the literature on this issue, with such a pattern of P4 intake, up to 30% of the women have inadequate levels of P4 when measured before the ET (10, 18, 27, 28). With the objective to increase serum P4 levels, Cédrin-Durnerin et al. (27) investigated the strategy to increase (twofold) the vaginal micronized P4 intake in women with low P4 values on the day of FET. In this specific study, increasing the dosage from 600 to 1,200 mg/day of micronized P4 did not improve the serum P4 concentration. Indeed, still, 30% of the women had P4 < 10 ng/ml, showing that there is probably a limit/problem in the absorption from the vaginal mucosa or a possible effect on the microbiome. Other authors did not manage to show an advantage of increasing the vaginal dosage of P4 (29, 30). However, previously, Alsbjerg et al. (31) described, in patients undergoing a FET in artificial cycles, better outcomes when vaginal P4 was doubled (from 90 mg vaginal gel once a day to twice a day).

To date, evidence on the most efficient way for P4 supplementation remains controversial. Subcutaneous P4 has been proven efficient for endometrial preparation and luteal phase support in both ART and FET cycles (18, 21). More specifically, subcutaneous (SC) P4 has been demonstrated to be associated with higher serum P4 levels than the vaginal route (30, 32), well accepted, and easily used by patients (33).

However, the unresolved question stays unanswered: is one assessment of serum P4 level enough to detect P4 deficiency in the luteal phase, given the very high variability of serum P4, and given the high correlation found between p- values and pregnancy rates? The current analysis shows that one serum P4 assessment is not enough to figure out the P4 trend within the luteal phase. According to the ROC analysis, we cannot be confident with a good serum P4 around the FET. Despite P4 **≥** 10.6 ng/ml the day before FET, the chance for P4-hCG < 10.6 ng/ml remains high and according to this study with a negative impact on LBR.

The retrospective nature of the study represents one of the most relevant limitations due to the unknown population bias. Furthermore, the decision of whether to supplement or not was arbitrarily taken by the clinicians. However, the feasibility of a randomized trial on this topic may have introduced ethical issues, given the proven beneficial effects of P4 supplementation. Furthermore, the sample size was not a particular limitation, except for subgroup C (low P4-hCG, not supplemented); the P4 assessment was performed in the morning for the entire population included. H owever, it is important to keep in mind the P4 variation, and LBR was calculated as the total number of live births on the number of positive hCG (hCG **≥** 10 IU) because only clinical pregnancy was included in the second part of the analysis. T herefore, the percentage should be regarded cautiously. Lastly, only cycles supplemented with vaginal P4 were included in the final analysis, and P4 was not assessed after the supplementation.

However, this is a single-center study. T herefore, the major strength is that it was conducted under the same laboratory and clinical conditions. The double determination of P4 the day before FET and on the day of the pregnancy test allows an excellent opportunity for developing a “rescue protocol” even in those cases with adequate serum P4 around the FET, providing an individualized strategy based on each patient’s situation. Lastly, a rigorous analysis was performed to better understand the causes of the influence of P4 on LBR by considering the hCG values and their possible impact on LBR.

## Conclusions

The results of our studies point consistently in the same direction, i.e., that P4 should be measured repeatedly in the luteal phase of an artificial cycle and, in case of inadequate levels, supplemented.

If measuring P4 at one timepoint could already be seen as personalization of care, repeated P4 assessment represents a step forward to account for the variability of the clinical situation in that specific critical period. We should not be 100% confident about adequate P4 levels around the ET, as this is not a guarantee of adequate P4 levels on the day of the pregnancy test and can compromise the pregnancy results. Finally, larger and more robust confirmatory studies are warranted to validate the present findings.

## Data availability statement

The raw data supporting the conclusions of this article will be made available by the authors, without undue reservation.

## Ethics statement

The studies involving human participants were reviewed and approved by institutional Review board of Dexeus Mujer (approval number: 072020102604). The patients/participants provided their written informed consent to participate in this study.

## Author contributions

CB conceived the original idea and the overall design of the study. GS and IR performed the statistical analysis. RA, AM, PN, and CB made substantial contributions to the acquisition and interpretation of data and critically assessed the results in the context of scientific literature. RA wrote the article. G-FI and PN revised the final draft of the manuscript. All the authors substantially revised the manuscript, have approved the submitted version, and have agreed both to be personally accountable for the authors’ own contributions and to ensure that questions related to the accuracy or integrity of any part of the work, even ones in which the authors were not personally involved, were appropriately investigated and resolved and that the resolution is documented in the literature.
